# The transmembrane IL-15 isoform expressed on human melanoma cells triggers modulatory effects on tumor progression upon stimulation with the soluble IL-15Rα chain

**DOI:** 10.3389/fimmu.2026.1798481

**Published:** 2026-04-15

**Authors:** Sergio Forcelloni, Julien Giron-Michel, Piero Del Boccio, Maria Concetta Cufaro, Alice Di Sebastiano, Francesca Romana Mariotti, Cecilia Ciancaglini, Salem Chouaib, Mael Padelli, Simone Vespa, Giang Dang Mac, Stefan Ebert, Stéphanie Buart, Enrico Maggi, Lorenzo Moretta, Paola Vacca, Nicola Tumino, Linda Quatrini, Ignazio Caruana, Bruno Azzarone, Silvia Santopolo

**Affiliations:** 1 Innate Lymphoid Cells Research Unit, Bambino Gesù Children’s Hospital, IRCCS, Rome, Italy; 2 INSERM UMRS 1197, Hôpital Paul Brousse, Villejuif Cedex, France; 3 Proteomics and Metabolomics Core Facility, Center for Advanced Studies and Technology (CAST), “G. d’Annunzio” University of Chieti-Pescara, Chieti, Italy; 4 Department of Science, “G. d’Annunzio” University of Chieti-Pescara, Chieti, Italy; 5 Department of Innovative Technologies in Medicine and Dentistry, “G. d’Annunzio” University of Chieti-Pescara, Chieti, Italy; 6 Department of Neuroscience Imaging and Clinical Sciences, “G. d’Annunzio” University of Chieti-Pescara, Chieti, Italy; 7 Tumor Immunology Research Unit, Bambino Gesù Children’s Hospital, IRCCS, Rome, Italy; 8 Institut National de la Santé et de la Recherche Médicale Unitè Mixte Recherche (INSERM UMR) 1186, Integrative Tumor Immunology and Cancer Immunotherapy, Gustave Roussy, École Pratique des Hautes Études (EPHE), Faculty De Médecine Univ. Paris-Sud, University Paris-Saclay, Villejuif, France; 9 Thumbay Research Institute for Precision Medicine, Gulf Medical University, Ajman, United Arab Emirates; 10 Department of Biochemistry and Oncogenetics, Paul Brousse Hospital, AP-HP, Villejuif, France; 11 Department of Pediatrics, Hematology, Oncology, Stem Cell Transplantation and Cell Therapy, University Hospital Würzburg, Würzburg, Germany

**Keywords:** cytokine, IL15, IL15Rα, natural killer (NK) cells, skin cancer (melanoma)

## Abstract

Interleukin-15 (IL-15) is expressed in various cancers, including melanoma, where it exists in distinct membrane-associated isoforms. Primary melanoma cells predominantly express the non-cleavable transmembrane form (tmbIL-15), while metastatic cells also express a cleavable membrane-bound form (mbIL-15) complexed with IL-15Rα. As tmbIL-15 is capable of reverse signaling upon IL-15Rα engagement, we investigated how this signaling axis modulates melanoma cell behavior across tumor stages. Transcriptomic analysis of melanoma patients revealed that high IL-15 expression correlates with immune activation, inflammation and epithelial-to-mesenchymal transition (EMT), along with coordinated upregulation of IL-15 receptor subunits. Proteomic profiling of melanoma cell lines stimulated with soluble IL-15Rα (sIL-15Rα) uncovered distinct, stage-specific responses. Although several proteins were commonly deregulated across cell lines, most showed opposite regulation in primary versus metastatic models, indicating that tmbIL-15 reverse signaling triggers context-dependent programs influenced by tumor progression. A stringent cross-comparison identified five proteins (PSAP, MARCKS, eEF1A1, DDX39B, and RACK1) as consistently and differentially regulated across tumor stages. Further comparison with published NK cell co-culture and EMT cytokine stimulation datasets revealed a subset of shared effectors, notably PSAP, TPM3 isoform 2 and MARCKS, suggesting that IL-15Rα–induced tmbIL-15 signaling is part of the immune editing phenomenon eliciting pro-tumoral activities complementary to the EMT process. Among these, PSAP emerged as the most robustly and consistently modulated effector, upregulated in primary melanoma cells and downregulated in metastatic ones upon sIL-15Rα stimulation. Its expression correlated positively with CD8+ T cell infiltration and negatively with NK cell infiltration, with distinct transcriptomic programs associated with high PSAP expression in primary versus metastatic settings. Altogether, these findings identify PSAP as a stage-specific mediator of tmbIL-15 reverse signaling in melanoma, integrating immune and EMT-related cues with potential implications for tumor progression and microenvironmental remodeling.

## Introduction

Melanoma is the most aggressive form of skin cancer. While it only accounts for about 4% of all skin cancer cases, it causes nearly 50% of related deaths worldwide (1, 2). Early-life exposure to sunlight, particularly ultraviolet B (UV-B) radiation from sunlight, is a major risk factor, contributing to approximately 60–70% of cutaneous malignant melanomas (3, 4). UV-B not only induces mutagenic DNA damage and oxidative stress but also promotes chronic inflammation milieu by recruiting immune cells and triggering the release of pro-inflammatory cytokines. This sustained inflammatory microenvironment may promote tumor initiation and progression (5–8). Among these cytokines, Interleukin-15 (IL-15), was shown by Mohamadzadeh et al. to be upregulated in keratinocytes and melanocytes upon UV-B exposure (9, 10), suggesting a potential role in melanomagenesis. IL-15 is a 14–15 kDa four-helix bundle cytokine critical for the development and homeostasis of NK, NKT, and CD8+ T cells. It is produced by a wide range of cells, including monocytes, dendritic cells, fibroblasts and epithelial cells, both in healthy and neoplastic tissues. In normal skin, IL-15 is constitutively expressed by basal keratinocytes and melanocytes, where it supports the maintenance of tissue-resident memory T cells (Trm) (11).

IL-15 is aberrantly expressed in melanocytic lesions, with high intratumoral IL-15 and IL-15Rα levels correlating with poor overall survival in stage IV melanoma patients (11). Moreover, UV-induced IL-15 upregulation in nevi may precede mutational events, suggesting it acts as an early mediator of melanoma development (12, 13).

Several functional isoforms of IL-15 have been identified. i) The soluble monomeric form is secreted at low levels due to multiple retention mechanisms (14, 15). IL−15 activates immune cells via an intermediate−affinity heterodimer receptor (IL−2Rβ/γc), shared with IL−2. Association with the high−affinity IL−15−specific IL−15Rα chain confers specificity and enhances signal transduction. ii) A membrane-bound form complexed with IL-15Rα (mbIL-15/IL-15Rα) remains at the cell surface and can signal to IL−2Rβ/γc-expressing cells either on neighboring cells (trans-presentation) or on the same cell (cis-presentation) (16, 17). iii) This complex may be released as a soluble IL-15/IL-15Rα form (sIL-15/IL-15Rα) either by direct secretion or by ADAM17-mediated cleavage (13, 18, 19). Compared to monomeric IL-15, the soluble complex has greater stability and potency, earning the designation “IL-15 superagonist” (18, 19). iv) A distinct transmembrane isoform (tmbIL-15) is anchored via an N-terminal hydrophobic sequence (aa 21–41) (20–23). Unlike mbIL-15, it is not cleavable and mediates bidirectional signaling: trans-presentation to IL-15Rβγ- or IL-15Rαβγ-expressing cells, and reverse signaling upon engagement by soluble or membrane IL-15Rα (20–22).

We previously demonstrated that two membrane-associated isoforms of IL-15 are differentially expressed in melanoma: primary tumors predominantly express tmbIL-15, whereas metastatic cells co-express both tmbIL-15 and the cleavable mbIL-15 isoform (11). In the above-mentioned study, we showed that membrane-bound and soluble IL-15/IL-15Rα complexes persist throughout melanoma progression and shape the immune landscape in a stage-dependent manner. While these cleavable forms promote cytotoxic NK and T cell responses at early stages, they paradoxically support the emergence of anergic, dysfunctional NK cells in advanced disease (11). The present study focuses on the tmbIL-15 isoform, expressed in both primary and metastatic tumors, to investigate how reverse signaling, triggered by soluble IL-15Rα, modulates melanoma cell-intrinsic programs and potentially contributes to tumor progression.

## Results

### High IL-15 expression is associated with immune and EMT-related pathways modulation and correlates with IL-15 receptor upregulation in melanoma

**Figure 1:**
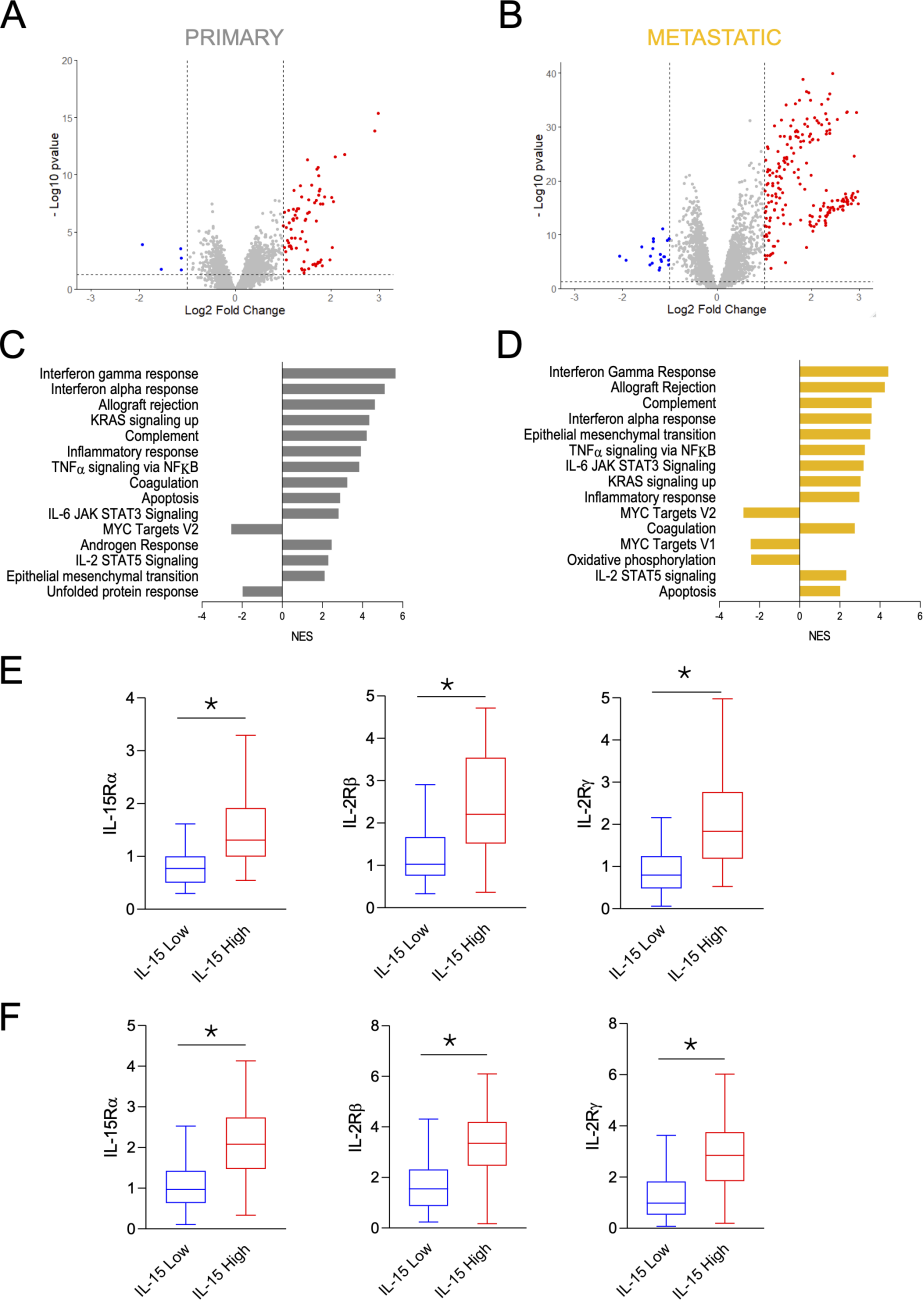
Transcriptomic analysis in primary and metastatic melanoma patients stratified by IL-15 expression levels. **(A, B)** Volcano plots of the differentially expressed genes (DEG) in IL-15 high versus IL-15 low in primary **(A)** and metastatic **(B)** melanoma patients in the TCGA Melanoma SKCM data set. Samples were divided into categories of high and low IL-15 expression by median as a cut-off value. Vertical lines indicate |log2 Fold Change|≥1 and the horizontal line indicates p ≤ 0,05 representing cut-off lines applied to filter significant DEGs. Not significant DEGs are represented in grey, significant upregulated and downregulated DEGs are represented respectively in red or in blue. **(C, D)** Bar plots showing normalized enrichment scores (NES) of the deregulated hallmark pathways between IL-15 high versus IL-15 low in primary **(C)** and metastatic **(D)** tumors in the TCGA Melanoma SKCM data set. E-F Box plots showing IL-15Rα, IL-2Rβ and IL-2Rγ expression (log2FPKM-UQ+1) in IL-15 low (blue) and IL-15 high (red) primary **(E)** and metastatic **(E, F)** patients in the TCGA Melanoma SKCM data set. *p ≤ 0,05 Welch’s t-test.

IL-15 is expressed in multiple cancers, including melanoma, where it influences survival in a stage-dependent manner, partly through effects on NK cell activity (2, 11). Primary melanoma cells express only the tmbIL-15, whereas metastatic melanoma cells also express a PMA-cleavable mbIL-15 form (11). To better understand the consequences of these isoform differences, we analyzed data from the publicly available TCGA (The Cancer Genome Atlas) Melanoma (SKCM) dataset, stratifying primary and metastatic melanomas by IL-15 expression level (primary: IL-15 high n = 52, low n = 52; metastatic: IL-15 high n = 185, low n = 185) (**Figures 1A, B**) (24). Consistent with literature indicating that IL-15 expression regulates EMT transition in various cancers, high IL-15 expression was associated with a strong enrichment in EMT gene expression in metastatic melanoma (GSEA enrichment p = 0,00058), and a weaker but significant enrichment in primary tumors (GSEA enrichment p = 0,0352) (**Figures 1C, D**). In both primary and metastatic tumors, high IL-15 expression was associated with upregulation of interferon and inflammatory pathways, apoptosis, KRAS, IL-6 and TNFα signaling, as well as downregulation of MYC targets. Unique patterns were observed by stage: unfolded protein response was reduced in primary IL-15 high tumors, while oxidative phosphorylation was decreased in metastatic IL-15 high tumors (**Figures 1C, D**).

Across both settings, high IL-15 expression correlated with higher expression of all three IL-15 receptor subunits, IL-15Rα, IL-2Rβ, IL-2Rγ (primary: p = 5,944e-8, p = 3,164e-7, p = 0,000022; metastatic: p = 6,046e-7, p = 6,993e-35, p = 2,623e-30), suggesting enhanced capacity to respond to IL-15 signaling (**Figures 1E, F**).

### Primary and metastatic melanoma cells differentially express cleavage-resistant transmembrane IL-15 and ADAM17-sensitive membrane-bound IL-15 isoforms

**Figure 2:**
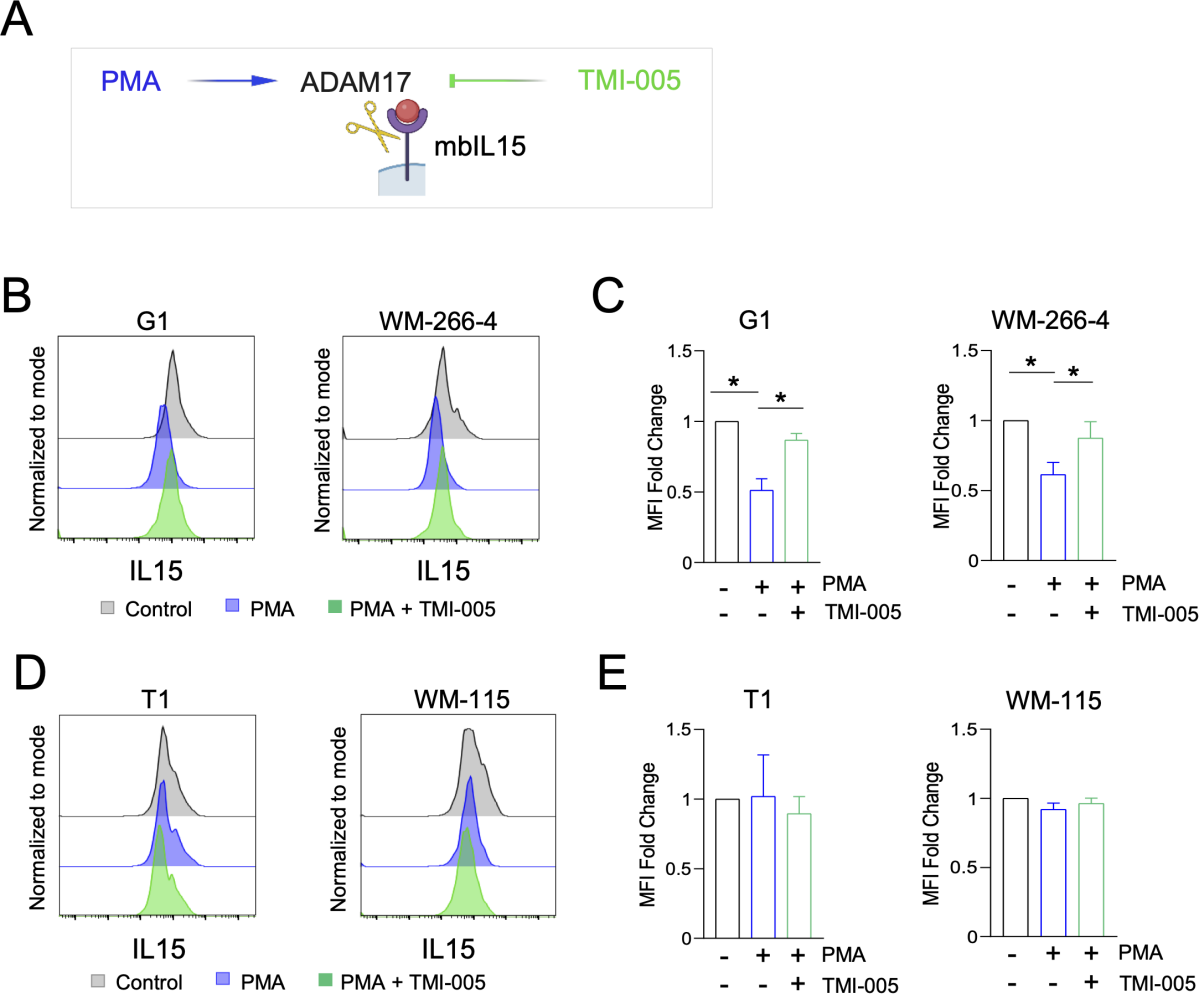
Analysis of membrane IL-15 isoforms expression in primary and metastatic melanoma cell lines. **(A)** Schematic representation of the mbIL-15 isoform cleavage by ADAM17, which is inhibited by TMI-005 and induced by PMA (created in BioRender). **(B–E)** Two pairs of primary/metastatic melanoma cell lines (T1/G1, WM-115/WM-266-4) were treated or not (Control, grey) with 100 ng/ml PMA alone (blue) or with 100 ng/ml PMA and 25μm TMI-005 (green) for 3h. **(B, D)** Flow-cytometry histograms of the membrane IL-15 expression from one representative experiment and **(C, E)** relative quantifications expressed as mean ± SD of three biological replicates are shown. *p ≤ 0,05 ANOVA test.

We previously demonstrated that primary melanoma cell lines (including T1 and WM−115) expressed only the PMA-insensitive tmbIL−15 isoform, while metastatic lines (including G1 and WM−266−4) expressed both tmbIL−15 and PMA−sensitive mbIL−15 (11). Since PMA activates the disintegrin and metalloproteinase-17 (ADAM17), we examined whether mbIL-15 cleavage in metastatic melanoma cell lines is ADAM17-dependent using the specific ADAM17 inhibitor TMI-005 (**Figure 2A**).

IL-15 expression was analyzed by flow cytometry in two primary/metastatic melanoma cell lines pairs, T1/G1 and WM-115/WM-266-4, each derived from the same patient. In accordance with our previous results, flow cytometry analysis confirmed that only the metastatic lines (G1 and WM−266−4) expressed PMA−sensitive mbIL−15, in addition to the tmbIL−15 isoform, as indicated by reduced IL−15 levels after PMA treatment (**Figures 2B, C**). Interestingly, ADAM17 inhibition by TMI-005 treatment (25µm) prevented PMA-induced mbIL-15 cleavage on metastatic cells (G1 and WM-266-4), demonstrating that mbIL-15 isoform is cleaved by ADAM17 (**Figures 2B, C**) (25). As expected, primary T1 and WM-115 cells that do not express the mbIL-15 isoform, showed no changes with PMA or TMI-005 (**Figures 2D, E**) (11).

### Stimulation with IL-15Rα triggers specific proteomic programs in primary and metastatic melanoma cells

**Figure 3:**
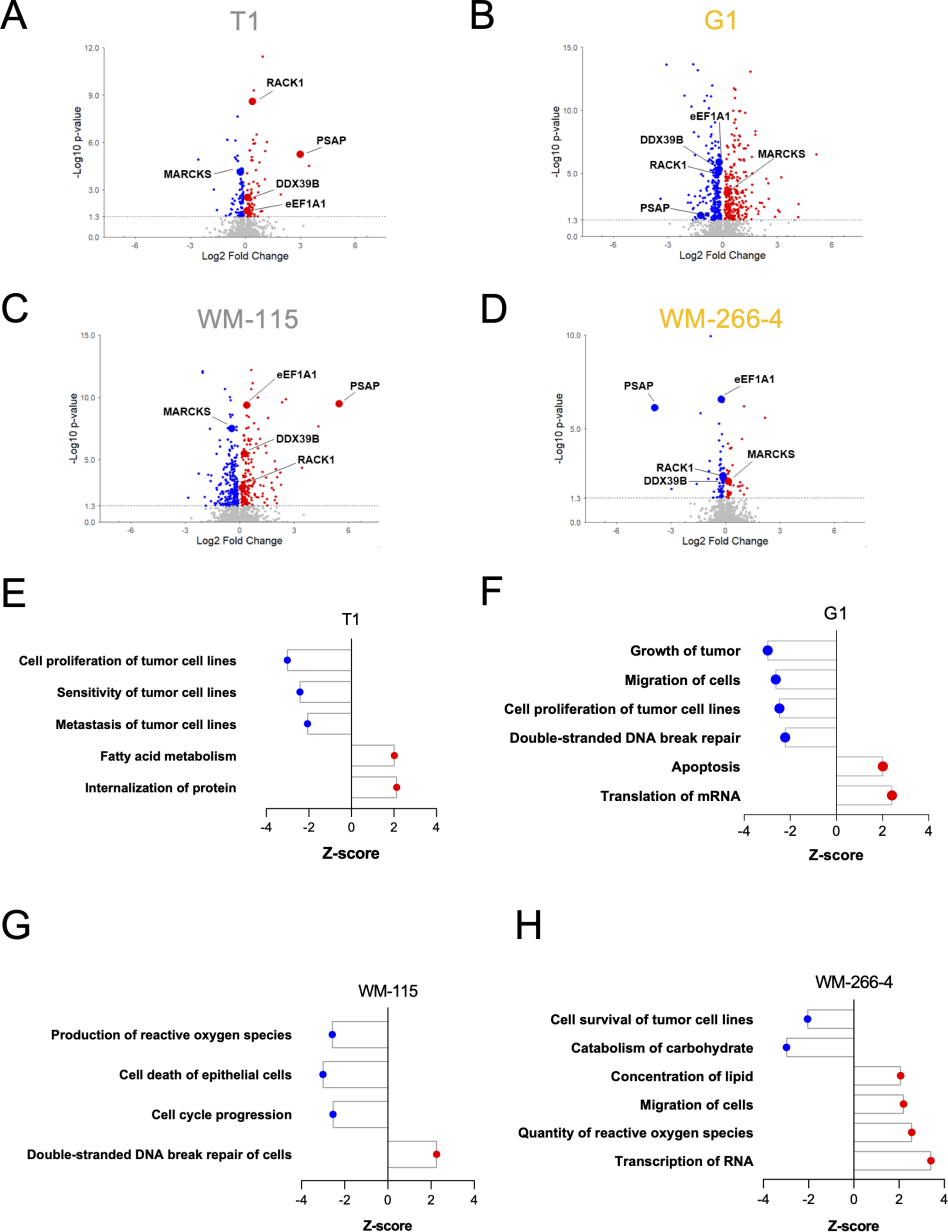
Proteomic profiling of primary and metastatic melanoma cell lines stimulated with soluble IL-15Rα. **(A–D)** Two primary/metastatic melanoma cell line pairs, T1 **(A)**, G1 **(B)** and WM-115 **(C)**, WM-266-4 **(D)** were stimulated with 10 ng/ml sIL-15Rα or not for 72h and quantitative proteomic differential analysis was performed between sIL-15Rα-stimulated and controls in each cell line (n=3). Volcano plots of the differentially expressed proteins are shown. Horizontal line indicates p ≤ 0,05 representing cut-off lines applied to filter significant deregulated proteins between sIL-15Rα-stimulated and control samples. Not significant proteins are represented in grey, significant upregulated and downregulated proteins are represented respectively in red or in blue. Selected proteins that are significantly differentially regulated across the four cell lines are highlighted and labelled in the volcano plots. **(E–H)** The entire protein ratio data sets were used for functional analysis by IPA (Ingenuity Pathway Analysis). Pathways with Z-scores ≤ -2 or ≥ 2 were respectively considered significantly inhibited (blue) or activated (red). **(A–D)** ANOVA test.

Given that both primary and metastatic melanoma express tmbIL-15, we used quantitative proteomics to assess global changes after soluble IL-15Rα (sIL-15Rα) stimulation. Paired primary/metastatic cell lines (T1/G1, WM-115/WM-266-4) were treated with recombinant human sIL-15Rα for 72 h (**Supplementary Figures S1A, B**). Significant protein deregulation was observed in each line with respect to the relative controls (p ≤ 0,05): 162 proteins in T1, 481 in G1, 421 in WM-115 and 100 in WM-266-4 (**Figures 3A–D**; **Supplementary Figure S1C**). The magnitude of response did not correlate with tumor stage or patient origin, suggesting cell line-specific variability.

To further explore the functional relevance of the proteins regulated upon sIL-15Rα stimulation, we performed pathway enrichment analysis for each cell line (**Figures 3E–H**). This revealed both common and distinct biological processes modulated by reverse signaling. In T1 primary cells, sIL-15Rα led to downregulation of pathways linked to proliferation, drug sensitivity and metastasis, alongside increased fatty acid metabolism and protein internalization, while in G1 metastatic cells, tumor growth, migration, proliferation and DNA double-strand break (DSB) repair were suppressed, but apoptosis and mRNA translation were upregulated. WM-115 cells showed reduced ROS production, cell death and cell cycle progression, together with enhanced DSB repair. By contrast, WM-266-4 cells displayed decreased survival and carbohydrate metabolism pathways, but increased lipid accumulation, migration, ROS levels and RNA transcription (**Figures 3E–H**). These results indicate that sIL-15Rα stimulation induces distinct proteomic programs depending on tumor stage and genetic background, yet recurrent themes such as metabolic rewiring, survival modulation and stress adaptation emerge across models.

### A core protein set reveals divergent tmbIL-15 reverse signaling in primary versus metastatic melanoma cells

**Figure 4:**
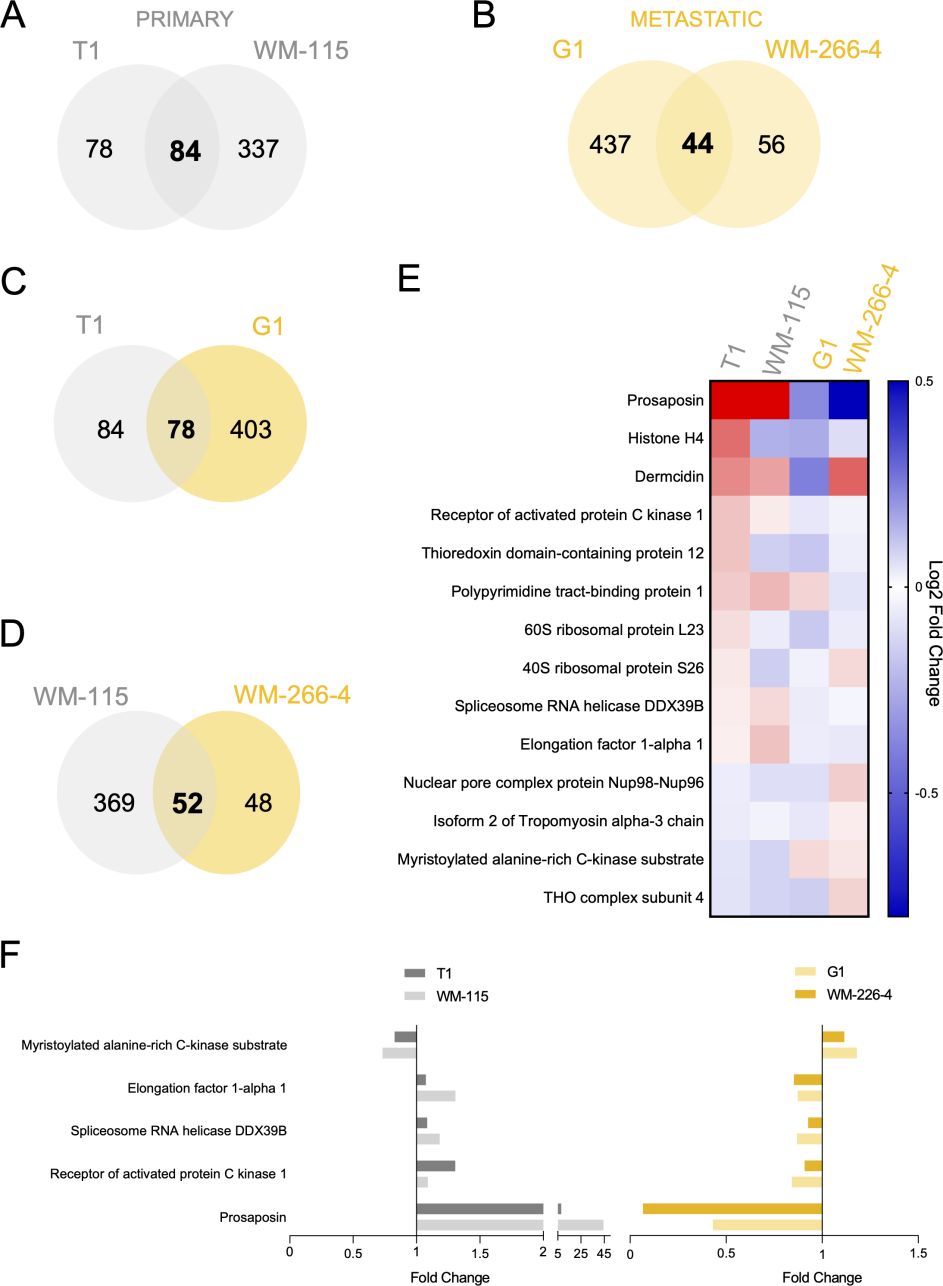
Shared and unique differentially expressed proteins among primary and metastatic melanoma cell lines after soluble IL-15Rα stimulation. **(A, B)** Venn diagrams showing the intersection of significant deregulated proteins after IL-15Rα stimulation between the two primary T1/WM-115 **(A)** and metastatic G1/WM-266-4 **(B)** melanoma cell lines. **(C, D)** Venn diagrams showing the intersection of significant deregulated proteins after sIL-15Rα stimulation between primary and metastatic cells in each primary/metastatic pair melanoma cell lines, T1/G1 **(C)** and WM-115/WM-266-4 **(D)**. **(E)** Heat Map of the 14 significant deregulated proteins after sIL-15Rα stimulation in all four cell lines analyzed. Each row represents a single protein, each column represents a cell line. The gradual color ranging from blue to red represents the Log2 Fold Change protein expression between sIL-15Rα-stimulated and control samples (Z-score). **(F)** Selected deregulated proteins which show consistent regulation within tumor type, primary (left panel) or metastatic (right panel), but opposite regulation between primary and metastatic cells are shown.

To assess the overlap in proteomic responses to IL-15Rα stimulation, we performed intersection analyses of differentially expressed proteins across the four melanoma cell lines. When grouped by tumor stage, 84 proteins were commonly deregulated across the two primary cell lines (T1 and WM-115), and 44 among the metastatic cell lines (G1 and WM-266-4) (**Figures 4A–B**).

78 deregulated proteins were shared instead between the patient-matched T1 (primary) and G1 (metastatic) lines, and 52 between WM-115 and WM-266-4 (**Figures 4C, D**). Despite this overlap, most shared proteins displayed an opposite expression trend between primary and metastatic lines from the same patient, as shown by a significant negative correlation in sIL-15Rα-stimulated over control fold changes values of each cell line (**Supplementary Figures S2A, B**). This indicates that tmbIL-15 reverse signaling elicits context-dependent responses shaped by tumor stage, even within identical genetic backgrounds.

Across all four lines, 14 proteins were commonly deregulated (**Supplementary Figure S2C**), with their expression patterns summarized in **Figure 4E**. To refine candidates most likely involved in stage-specific reverse signaling, we focused on proteins showing consistent regulation within primary or metastatic lines, but opposite regulation between the two groups. This stringent filtering yielded five key proteins (**Figure 4F**). These include myristoylated alanine-rich C kinase substrate (MARCKS), a regulator involved in cytoskeleton dynamics and motility (26); elongation factor 1-alpha 1 (eEF1A1), a key component of the translational machinery and modulator of apoptosis (27); spliceosome RNA helicase DDX39B, a spliceosome helicase regulating RNA splicing and cell cycle (28); receptor of activated protein C kinase 1 (RACK1), a scaffolding protein involved in signal transduction and cell adhesion (29) and prosaposin (PSAP), a lysosomal protein implicated in lipid metabolism, immune modulation and tumor invasion (30, 31). These effectors participate in key pathways related to migration, survival and gene regulation. Their opposite regulation suggests differential tmbIL-15 signaling programs in early versus advanced melanoma. Among the five proteins, PSAP emerged as the most robustly and consistently regulated protein across all the cell lines analyzed, upregulated in primary cells and downregulated in metastatic cells, highlighting its role as a potential stage-specific mediator of IL-15Rα-induced reverse signaling.

### IL−15Rα−induced proteomic changes partially overlap with those triggered by NK cells and EMT-inducing cytokines

**Figure 5:**
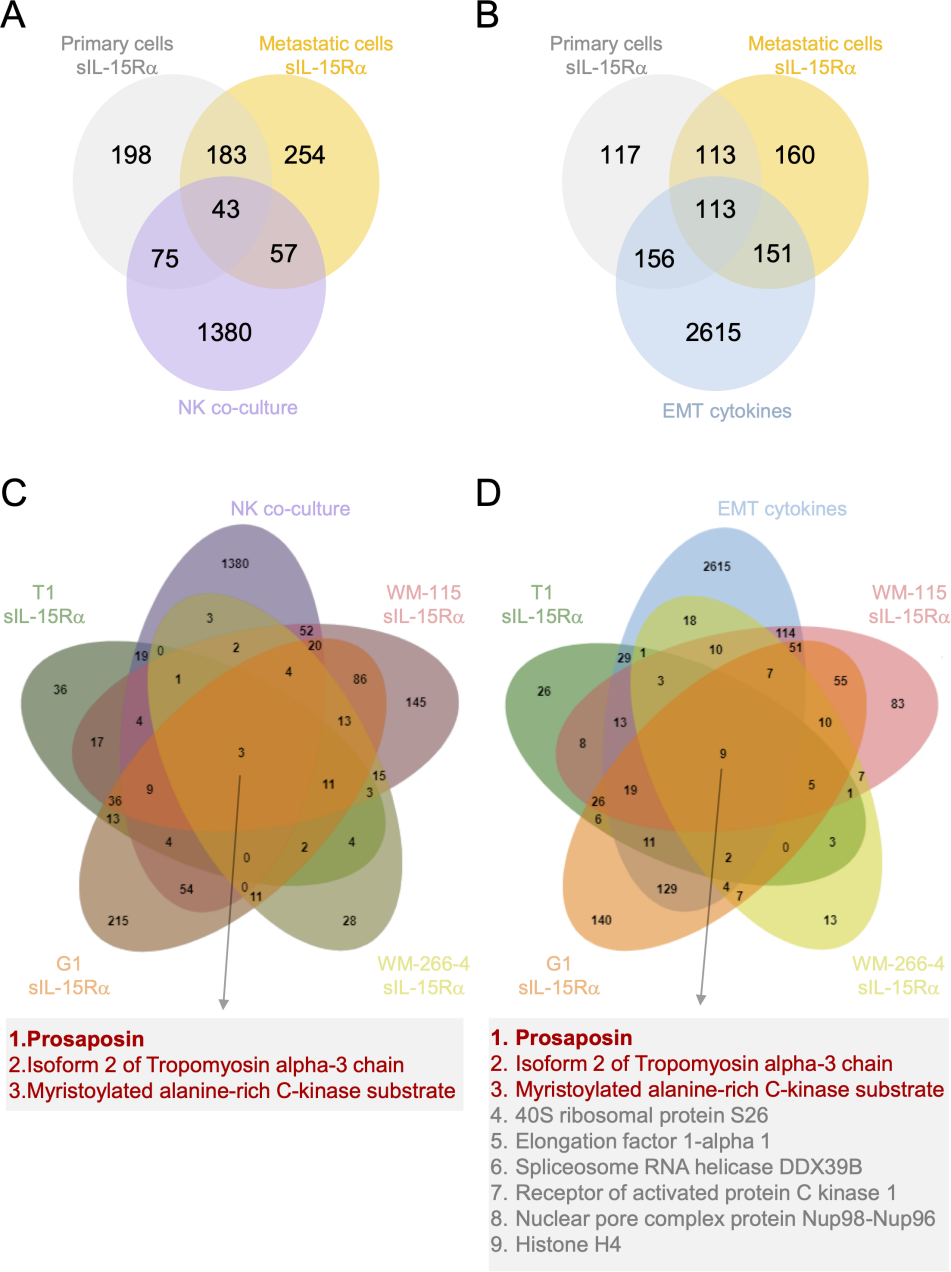
Comparative analysis of deregulated proteins in sIL-15Rα–stimulated and NK co-cultured or EMT-cytokine-treated melanoma cell lines. **(A, B)** Venn diagrams showing the intersection of significant deregulated proteins between sIL-15Rα stimulated primary (grey) and metastatic (yellow) melanoma cells with NK-cocultured [violet, **(A)**] or EMT-cytokines treated [blue, **(B)**] melanoma cells. **(C, D)** Venn diagrams showing the intersection of significant deregulated proteins between sIL-15Rα stimulated T1, G1, WM-115, WM-266–4 melanoma cells with NK-cocultured **(C)** or EMT-cytokines treated **(D)** melanoma cells. The list of the proteins commonly deregulated in all conditions is shown in the insets.

Since NK cells express membrane-bound IL-15Rα, we asked whether tmbIL-15 reverse signaling induced by sIL-15Rα stimulation recapitulates aspects of NK-mediated signaling. We compared our proteomic data with that of Huergo-Zapico et al. (32), where two melanoma cell lines (MeCoP and MeDeBO) were co-cultured with NK cells. This interaction induced an immune editing process leading to EMT. This analysis identified 175 shared deregulated proteins. Of these, 75 overlapped with our primary melanoma cell lines, 57 with metastatic cell lines and 43 with both (**Figure 5A**). These overlaps support the idea that tmbIL-15 may be functionally engaged by NK cell–derived IL-15Rα, mirroring the effect of sIL-15Rα in our system. As expected, the total number of deregulated proteins was higher in the NK co-culture dataset, reflecting the broader signaling complexity of cell-cell contact involving multiple surface receptors beyond IL-15Rα.

Given that the NK co-culture was shown to promote immune editing and induce EMT-like reprogramming (32), we next asked whether sIL-15Rα–mediated tmbIL-15 reverse signaling could also engage EMT-related pathways. To this end, we compared our proteomic data with the published dataset of melanoma cells stimulated with EMT-inducing cytokines (32).

We found 420 overlapping deregulated proteins: 156 specific to our primary lines, 151 to metastatic, and 113 common to both (**Figure 5B**). This strong convergence indicates that sIL-15Rα stimulation engages pathways also activated by cytokines driving EMT, consistent with IL-15’s proposed role in EMT modulation in other tumor types. Despite this extensive overlap, many differentially expressed proteins remained specific to each condition. As noted by Huergo-Zapico et al., the two melanoma cell lines in their study responded differently to NK contact or EMT- cytokines, underscoring the importance of cell-intrinsic factors (32). To address this, we refined our analysis by intersecting the NK and EMT datasets with each of our four melanoma lines, rather than grouping them by tumor type (primary or metastatic). This approach allowed us to focus the results on a small set of proteins consistently deregulated across all our models.

In the comparison with NK-coculture, only three proteins were commonly deregulated across all four lines: PSAP, Isoform 2 of Tropomyosin alpha-3 chain (TPM3) and Myristoilated alanine-rich C-kinase substrate (MARCKS) (**Figure 5C**). In the EMT-cytokine overlap, nine proteins were shared: PSAP, TPM3, MARCKS, 40S ribosomal protein S26 (RPS26), Elongation factor 1-alpha 1 (eEF1A1), DDX39B, Receptor of activated protein C kinase 1 (RACK1), Nuclear pore complex protein Nup98-Nup96 and Histone H4 (**Figure 5D**). Notably, PSAP, TPM3 and MARCKS were consistently regulated across all three conditions (sIL-15Rα stimulation, NK co-culture and EMT-cytokine exposure) highlighting them as central effectors at the intersection of IL-15 reverse signaling, immune editing and EMT reprogramming. This convergence suggests that tmbIL−15 reverse signaling engages a limited set of conserved effectors integrating immune, migratory, and EMT-related cues.

### Prosaposin is differentially regulated by tmbIL−15 reverse signaling and correlates with tumor progression and immune remodeling

**Figure 6:**
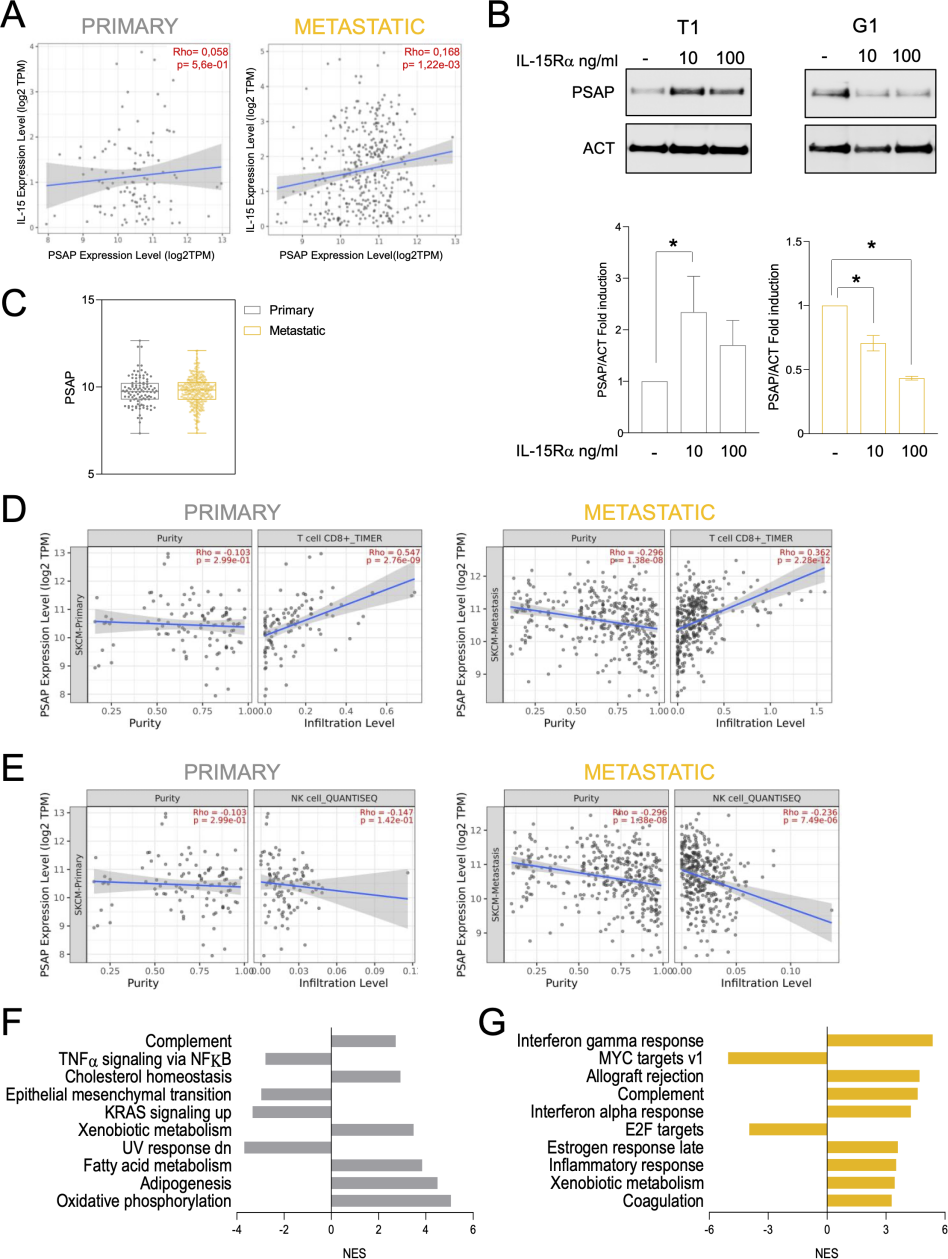
Regulation of prosaposin expression in sIL-15Rα–stimulated primary and metastatic melanoma cell lines and its correlation with immune cell infiltration and signaling pathways in melanoma patients’ datasets. **(A)** Scatterplots showing the correlation between IL-15 and PSAP expression levels in primary (left panel) and metastatic (right panel) tumors in the TCGA Melanoma SKCM data set. Rho and p values were determined by the Spearman correlation test. Linear regression and standard deviation are shown. **(B)** Western blot analysis of PSAP and β-Actin (ACT) protein levels in whole cell extracts from primary T1 (left panel) and metastatic G1 (right panel) melanoma cells treated with sIL-15Rα (10 AND 100 ng/ml) or not (Control) (upper panels). Quantification of the ratio between PSAP and ACT levels in the samples described above, expressed as mean ± SD of three biological replicates. *p ≤ 0,05 (ANOVA test) (lower panels). **(C)** Box plot showing PSAP gene expression levels in primary (grey) and metastatic (yellow) tumors in the TCGA Melanoma SKCM data set. Each dot represents a melanoma patient. **(D, E)** Correlation analysis between PSAP expression and immune infiltration of CD8+T cells **(D)** and NK cells **(E)** in primary (left panels) and metastatic (right panels) melanoma tumors in the TCGA Melanoma SKCM data set. Rho and p values were determined by the Spearman correlation test. Linear regression and standard deviation are shown. **(F, G)** Bar plots show normalized enrichment scores (NES) of the deregulated hallmark pathways between PSAP high versus PSAP low in primary **(F)** and metastatic **(G)** tumors in the TCGA Melanoma SKCM data set.

Among the proteins consistently deregulated across sIL-15Rα stimulation, NK co-culture and EMT-inducing cytokine datasets, PSAP emerged as the most robust and stage-specific effector. It was upregulated in both primary melanoma cell lines and downregulated in their metastatic counterparts after sIL-15Rα stimulation (**Figure 4**). To assess its clinical relevance, we analyzed PSAP expression in the TCGA SKCM transcriptomic dataset. PSAP levels positively correlated with IL-15 expression in both primary and metastatic samples, reaching statistical significance only in metastases (**Figure 6A**), suggesting a functional link emerging during tumor progression.

To further validate the proteomic trends observed in our cell lines, we performed Western blot analysis of PSAP expression upon stimulation with sIL-15Rα. Western blot analysis validated the proteomic findings: sIL-15Rα stimulation at 10 and 100 ng/ml increased PSAP expression in T1 primary cells but decreased it in G1 metastatic cells, supporting a tumor stage-dependent regulation of PSAP by tmbIL-15 reverse signaling (**Figure 6B**). Interestingly, PSAP baseline mRNA levels did not differ significantly between primary and metastatic tumors (**Figure 6C**), reinforcing the role of signaling context in its regulation.

We then examined the relationship between PSAP expression and immune infiltration in melanoma. PSAP levels positively correlated with CD8+ T cell infiltration in both tumor types (**Figure 6D**), but showed an inverse correlation with NK cell infiltration, significant only in metastatic tumors (**Figure 6E**). These associations point to a potential role for PSAP in shaping the immune landscape in a context-dependent manner.

To explore the functional impact of PSAP expression, we stratified TCGA samples into PSAP high and PSAP low groups (primary: n = 50/48; metastatic: n = 181/180) (**Supplementary Figures S3A, B**) and performed pathway enrichment analysis (**Figures 6F, G**). In primary tumors, high PSAP was associated with metabolic and homeostasis pathways (oxidative phosphorylation, fatty acid metabolism, cholesterol homeostasis) and with downregulation of inflammatory and invasive signatures (TNFα/NFκB, EMT, KRAS). Conversely, in metastatic tumors, high PSAP correlated with increased immune activation (IFNα/γ responses, inflammatory signaling, complement activation) and decreased proliferative programs (MYC, E2F targets). Together, these data suggest that PSAP contributes to anti-tumor features in both settings, but via distinct mechanisms: promoting a more stable, less invasive phenotype in primary tumors and enhancing immune activity in metastases. This dual behavior aligns with our *in vitro* data showing opposing PSAP responses to sIL-15Rα in primary versus metastatic cells, implying that tumor progression modulates the outcome of tmbIL-15 reverse signaling. Despite these functional associations, PSAP expression did not correlate with overall survival in either tumor group (**Supplementary Figures S3C, D**).

To further evaluate the impact of PSAP expression on the clinical outcome of melanoma patients treated with immune checkpoint inhibitors (ICI), we analyzed the Metastatic Melanoma (DFCI, Nat Med. 2019) dataset (33), restricting the cohort to patients who received ICI therapy. Kaplan Meier analysis revealed that high PSAP levels were significantly associated with higher progression-free survival (PFS) (**Supplementary Figures S4A–C**).

We then evaluated the impact of the additional tmbIL-15 reverse signaling-related key factors alone or in combination with PSAP expression. Interestingly, we found that low expression levels of eEF1A1, RACK1 and DDX39B were significantly associated with higher PFS, while the expression levels of MARCKS and TPM3 alone did not show a statistically significant association with PFS (**Supplementary Figure S4D**). Stratifying patients according to the mean expression of PSAP, eEF1A1, RACK1 and DDX39B (**Supplementary Figure S4E**) or PSAP, TPM3 and MARCKS (**Supplementary Figure S4F**) we found a significant correlation with PFS. Altogether, these findings support a potential link between tmbIL-15 reverse signaling and response to immunotherapy.

## Discussion

In the present study, we investigated the role of reverse signaling through the tmbIL-15 in melanoma progression (20, 21), building upon our previous work, which showed that tmbIL-15 expression in melanoma is stage-dependent: primary melanoma cells express exclusively the non-cleavable tmbIL-15, whereas metastatic cells co-express both tmbIL-15 and the cleavable mbIL-15 (11). We also showed that mbIL-15/IL-15Rα complexes at the melanoma cell surface can shape the immune landscape, initially enhancing cytotoxic T and NK cell activation but eventually contributing to NK cell dysfunction in advanced disease (11).

Herein, extending these observations, analysis of TCGA melanoma datasets stratified by IL-15 expression revealed that high IL-15 levels, in both primary and metastatic tumors, are positively associated with interferon signaling, inflammatory pathways, EMT induction, and increased IL-15 receptor subunit expression. These findings suggest the existence of a positive feedback loop that amplifies IL-15 activity at high levels and promotes the establishment of a pro-inflammatory tumor microenvironment (TME) favorable to melanoma progression.

Subsequently, we focused on the intrinsic effects of IL-15Rα-induced reverse signaling via tmbIL-15 in melanoma cells. Unlike classical cytokine signaling, this mechanism is independent of IL-15 secretion and is triggered by contact with IL-15Rα, either in soluble form or expressed at the surface of immune cells such as dendritic cells or NK cells.

Proteomic profiling of four patient-matched primary (T1, WM-115) and metastatic melanoma cell lines (G1, WM-266-4), revealed that sIL-15Rα stimulation elicits distinct molecular responses depending on tumor stage. Interestingly, despite partial overlaps between primary and metastatic proteomes, the directionality of protein regulation was often inverted between matched lines, supporting the concept that tumor progression reshapes tmbIL-15 reverse signaling outputs.

To refine the most relevant stage-dependent effectors, we identified a small set of proteins that were consistently deregulated across the two melanoma cell pairs (primary/metastatic). Among these, five showed inverse regulation between primary and metastatic contexts: PSAP (30, 31), MARCKS (34), eEF1A1 (27), DDX39B (28), and RACK1 (29). These proteins are involved in cell adhesion, cytoskeleton regulation, migration, melanoma-derived EVs trafficking, transcriptional control, and stress responses, functions critical to tumor progression, immune interaction and poor prognosis. Notably, cross-comparison with published proteomic datasets from melanoma cells exposed to NK cell co-culture or EMT-inducing cytokines (32) showed a strong overlap, pointing to a shared response axis between IL-15Rα engagement, immune editing, and EMT-like reprogramming. PSAP, TPM3 isoform 2, and MARCKS emerged as common effectors across all three conditions.

Among these, TPM3 isoform 2 is particularly interesting due to its known role in actin filament stabilization and cell motility. Tropomyosin family proteins are central to cytoskeletal dynamics, and TPM3 isoforms have been shown to influence processes such as wound healing, cancer cell invasion, and EMT (35). Isoform 2, specifically, has been implicated in enhancing mesenchymal traits in tumor cells, supporting increased plasticity and invasiveness. Its identification across sIL-15Rα stimulation, NK-induced immune editing, and EMT-mimicking conditions positions TPM3 as a molecular integrator of tumor motility and immune modulation.

MARCKS, another convergent effector, is a known substrate of PKC involved in actin cytoskeleton remodeling, membrane trafficking, and cell adhesion. Its overexpression and phosphorylation have been associated with increased motility and metastasis in various cancers, including cholangiocarcinoma and breast cancer (26). Moreover, MARCKS is highly expressed in immune cells, where it regulates inflammatory cytokine secretion and migratory behavior, suggesting it may also mediate crosstalk between melanoma cells and infiltrating immune populations.

PSAP stood out as the most consistently and strongly modulated protein. It was upregulated in primary melanoma cells and downregulated in metastatic ones upon sIL-15Rα stimulation, suggesting that reverse tmbIL-15 signaling is reprogrammed during tumor evolution. Functional enrichment analysis of PSAP high versus PSAP low tumors in the TCGA dataset revealed divergent transcriptional programs according to tumor stage. In primary tumors, high PSAP expression was associated with metabolic homeostasis and reduced inflammatory and invasive signaling (including EMT and KRAS pathways), whereas in metastatic tumors, PSAP-high status correlated with immune activation signatures, including type I and II interferon responses, and decreased proliferative markers. These observations are in line with a stage-specific dual role of PSAP, promoting differentiation and metabolic stability in early disease, and modulating immunogenicity in advanced tumors.

This context-dependent modulation is particularly compelling when considered alongside PSAP’s known roles in other cancers. Elevated PSAP levels are correlated with poor prognosis in glioblastoma, pancreatic, and gastric cancers (30, 31). Mechanistically, PSAP promotes tumor cell migration and invasion, in part by activating the TGF-β/Smad pathway, a key driver of EMT (36). It can also modulate the tumor microenvironment by regulating cytotoxic T cell infiltration (37), and it has been implicated in activating survival pathways such as PI3K/Akt and ERK in prostate and breast cancers, respectively (38, 39). Recent work by Sharma et al. has shown that PSAP glycosylation in tumor-infiltrating dendritic cells leads to its abnormal secretion, depleting lysosomal saposins, and impairing antigen processing thereby contributing to immune escape in melanoma (40). This reinforces the idea that PSAP is not merely a bystander, but rather an active regulator of both tumor cell behavior and immune interaction.

In this context, IL-15Rα-induced reverse signaling may modulate the immunogenicity of melanoma cells through PSAP, TPM3 and MARCKS, with opposite effects depending on tumor stage. This dual behavior aligns with the broader observation that EMT and immune evasion are interconnected: EMT programs not only promote motility and invasiveness but also downregulate MHC expression and increase resistance to cytotoxic lymphocytes. Hence, by modulating effectors such as PSAP, TPM3 and MARCKS, reverse tmbIL-15 signaling could contribute to tumor plasticity, enabling melanoma cells to dynamically shift from immune-responsive to immune-resistant phenotypes.

Altogether, our findings highlight reverse signaling through tmbIL-15 as a non-canonical mechanism by which melanoma cells integrate external cues, such as sIL-15Rα released from the tumor microenvironment or presented by immune cells, to regulate their own plasticity and immune interactions. By identifying shared effectors across sIL-15Rα stimulation, NK cell contact, and EMT signals, we propose a convergent pathway that governs both tumor cell phenotype and immune escape. The identification of PSAP, TPM3 isoform 2, and MARCKS as central components of this axis provides a rationale to further explore their potential as biomarkers or therapeutic targets, particularly in strategies aiming to re-sensitize tumors to immune responses or to block EMT-associated progression. Finally, our results suggest that tmbIL-15 reverse signaling may contribute to shaping the clinical outcome of melanoma patients receiving ICI. Despite relying on melanoma cell line models and integrative analyses of TCGA datasets, this study identifies a consistent set of candidate effectors associated with tmbIL-15 reverse signaling. Future studies combining *in vivo* models and mechanistic dissection of this pathway will be required to further establish its causal role in melanoma progression and immune modulation.

## Materials and methods

### Cell lines culture and treatments

The primary/metastatic cell lines pair WM-115/WM-266–4 (derived from the same patient) were purchased from the American Type Culture Collection (ATCC, Virginia, USA). The primary T1 and the metastatic melanoma G1 cell lines, derived from the same patient, were obtained from the Institute Gustave Roussy (Villejuif, France). The abovementioned cell lines were expanded in RPMI-1640 medium (Euroclone, Italy) supplemented with 2 mM L-glutamine (Euroclone), 1% streptomycin and penicillin (Euroclone), and 10% fetal bovine serum (FBS; Thermo-Fisher Scientific, Massachusetts, USA) at 37 °C with 5% CO2 (41). All cell lines were confirmed as mycoplasma-negative by reverse transcription PCR (RT-PCR) (Eurofins). In the experiments for IL-15 isoform characterization, melanoma cell lines were treated with 100 ng/ml of phorbol-12-myristate-13-acetate (PMA, Sigma-Aldrich) with or without 25μM TMI-005 (Apratastat, Sigma-Aldrich) for 3h as previously described (11, 42). In the experiments for quantitative proteomic analysis and western blot, melanoma cell lines were treated with 10 ng/ml of soluble recombinant human IL-15Rα (R&D) for 72h.

### TCGA data analysis

TCGA (The Cancer Genome Atlas) SKCM data were obtained from the UCSC Xena resource (43). For gene expression analysis, the RNAseq data were downloaded as FPKM-UQ+1 values. The differentially expressed genes (DEGs) were then refined based on the criteria of |log2FoldChange|≥1 and pvalue ≤ 0,05. Results were visually represented in volcano plots using the ggplot2 package in R. Hallmark and canonical pathway analysis of the data from the TCGA SKCM data set (43, 44) were performed using Gene Set Enrichment Analysis (GSEA) and enrichment scores were calculated using a weighted Kolmogorov-Smirnov-like statistic while pvalues were calculated through permutation testing.

Immune infiltration levels in PSAP high and PSAP low groups from TCGA SKCM patients were evaluated using TIMER 2.0 (44). Overall survival (OS) was analyzed using UCSC Xena resource in the TCGA SKCM data (43). Progression free survival was analyzed using Kaplan Meier Plotter in the Metastatic Melanoma (DFCI, Nat Med. 2019) dataset (33).

The Kaplan Meier plots were generated based on the expression profiles, which were categorized into low and high expression groups according to the median expression value or the optimal percentile-based cut-off. Survival differences were evaluated using the log-rank test.

### Flow cytometry

For the detection of membrane IL-15 isoforms, melanoma cells were stained with the anti-human IL-15 antibody (R&D) for 15 minutes at 4 °C. Cells were acquired on a CytoFLEX LX flow cytometer (Beckman Coulter). At least 10,000 events for each condition were analyzed (45, 46). Dead cells were excluded using LIVE/DEAD Fixable Blue Dead Cell Stain Kit (Invitrogen) (47).The acquired data were analyzed using CytExpert-2.3 (Beckman Coulter) and FlowJo v.10 software (BD Biosciences).

### Label free proteomics

Label-free proteomics was performed on eight experimental conditions: four melanoma cell lines (G1, T1, WM-115 and WM266-4), each stimulated or not with soluble IL-15Rα. Samples were lysed in lysis buffer (6 M urea in 100 mM Tris/HCl, pH 7.5), followed by ultrasonication (80% amplitude, Sonicator U200S control, IKA Labortechnik, Staufen, Germany) on ice. A total of 25 µg of protein per sample condition was subjected to enzymatic digestion using trypsin (0.5 µg/µl, Sigma-Aldrich, St. Louis, MI, USA), following the SP3 (Single-Pot Solid-Phase-enhanced Sample Preparation) protocol. Proteins were initially reduced with 1 M dithiothreitol (DTT, Merck, D0632) and alkylated with 1 M iodoacetamide (IAA, Merck, I6125), both at a final concentration of 100 mM (sample volume/10), with 30 minutes incubations on ice (DTT) and in the dark (IAA). Protein binding to magnetic beads was carried out using a 1:10 protein-to-bead mass ratio. Beads consisted of a 1:1 mixture of two types of Sera-Mag SpeedBeads (Carboxylate-modified particles E3 and E7; Thermo Fisher: 65152105050250 and 45152105050250), each at a concentration of 15 µg/µl. Ethanol was added to the protein-bead mixtures at a 1:1 (v/v) ratio to reach a final ethanol concentration of 100%. Following binding, three consecutive washes with 180 µl of 80% ethanol were performed. For on-bead digestion, samples were resuspended in 200 mM ammonium bicarbonate containing sequencing-grade trypsin at a 1:50 enzyme-to-protein ratio (w/w) and then incubated at 37 °C for 14 hours in a final volume of 100 µl. Upon completion of digestion, samples were magnetically separated and the resulting peptide-containing supernatants were collected. Data acquisition was performed by analyzing 2 µl of tryptic peptides from each condition in triplicate with an EASY-spray AcclaimTM PepMapTM C18 (75 µm ID, 15 cm L, 2 µm PS, Thermo Fisher Scientific) nanoscale chromatographic column and a total run time of 65 minutes using a chromatographic gradient from 5 to 90% of ACN. The mass spectrometry proteomics data have been deposited to the ProteomeXchange Consortium via the PRIDE (48) partner repository, with the dataset identifier PXD076097. Proteomics raw data were processed using Thermo Proteome Discoverer (PD) version 2.4.0.305 (Thermo Fisher) to identify differentially expressed proteins (DEPs) between sIL-15Rα-stimulated and control conditions for each of the four melanoma cell lines (G1, T1, WM-115 and WM-266-4), using the human proteome database (UniProt ID: 9606). Protein ratios were calculated as “Protein Abundance Based” and the statistics hypothesis by ANOVA was based on the abundances of individual proteins or peptides. To ensure robust protein quantification, data were filtered by applying a high-confidence threshold at the protein level (FDR 0,01) and requiring a minimum of two unique peptides per protein. Common contaminants were excluded from the analysis.

### Ingenuity pathway analysis

Gene Ontology (GO) functional enrichment analysis was conducted using Ingenuity Pathway Analysis (IPA) (Qiagen) software to identify significantly enriched biological pathways associated with the proteomic profile. IPA can predict the activation (Z-scores ≥ 2,0) or inhibition (Z-scores ≤ −2,0) of canonical pathways in the loaded proteins dataset based on published literature. Functional annotation significance was assessed by pvalue, with values < 0,05 considered statistically significant.

### Western blot analysis

Cells were washed twice with ice-cold PBS and then lysed in lysis buffer as previously described (49). Equal amounts of proteins (20 μg) were separated by SDS polyacrylamide gel electrophoresis (PAGE) and transferred onto nitrocellulose membranes as described (50). Membranes were then incubated with rabbit polyclonal anti-PSAP (Proteintech) or monoclonal anti-β-Actin (Sigma-Aldrich) antibodies, followed by decoration with peroxidase-labeled anti-rabbit or anti-mouse IgG (Super-Signal detection kit, Pierce) (51). The results shown are representative of three independent experiments.

### Statistical analysis

Statistical analysis was performed using GraphPad Prism software version 8.0.1. Comparisons among groups were performed by one-way ANOVA with Bonferroni adjustments or Welch’s t-test. Data are expressed as the means ± standard deviations (SD) of triplicate samples. For correlation analyses, the Pearson or Spearman test were used. p ≤ 0,05 was considered significant.

## Data Availability

The mass spectrometry proteomics data have been deposited to the ProteomeXchange Consortium via the PRIDE (48) partner repository with the dataset identifier PXD076097.
