# Supplementary material for: The transmembrane IL-15 isoform expressed on human melanoma cells triggers modulatory effects on tumor progression upon stimulation with the soluble IL-15Rα chain
Source: Front Immunol. 2026 Apr 15;17:1798481. doi: 10.3389/fimmu.2026.1798481 (PMC13124470; doi:10.3389/fimmu.2026.1798481)
Supplement: Supplementary file 1 [file DataSheet1.pdf]

Supplementary Figures

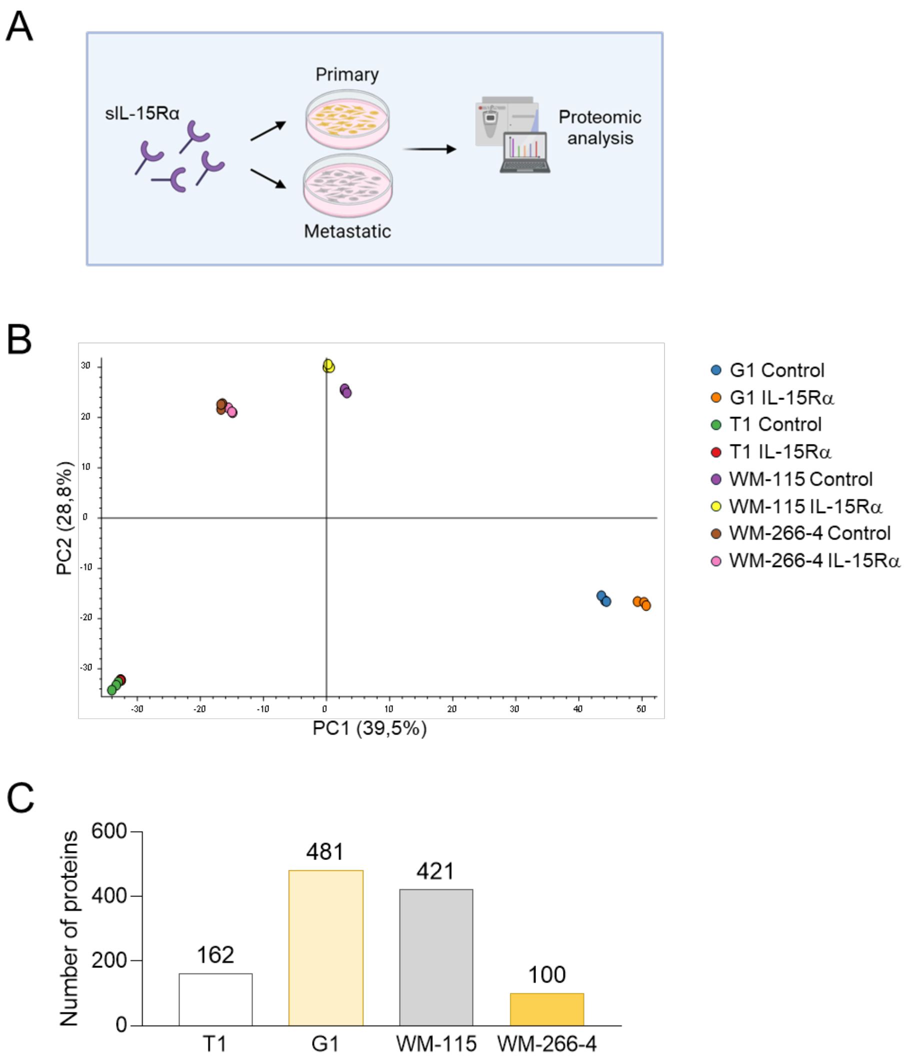

Figure Supplementary 1. Proteomic analysis overview of primary and metastatic melanoma cell lines stimulated with soluble IL-15Rα

A Schematic representation of the quantitative proteomic analysis on primary (T1, WM-115) and metastatic (G1, WM-266-4) melanoma cell lines treated or not with 10 ng/ml sIL-15Rα for 72h, created in BioRender. **B** Principal Component Analysis (PCA) for the quantitative proteomic analysis described in A (n=3). **C** Total number of significant deregulated proteins (p≤0,05) in the quantitative proteomic differential analysis between IL-15Rα-stimulated and control samples in each cell line.

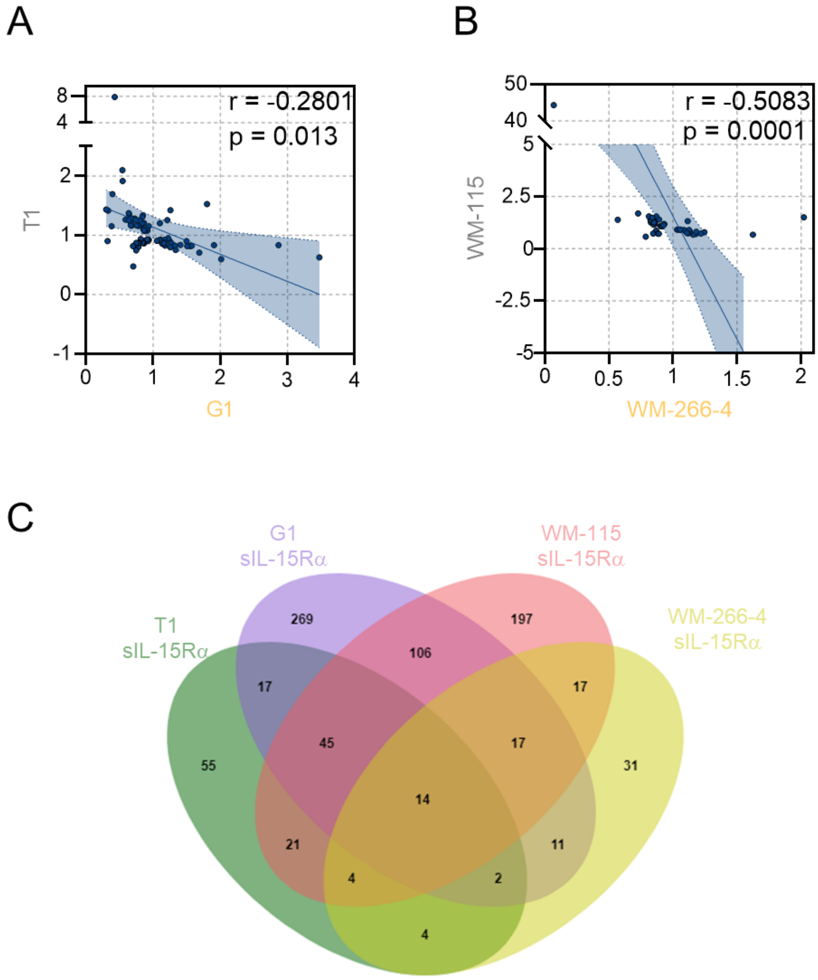

Figure Supplementary 2. Overlap of differentially expressed proteins across primary and metastatic melanoma cell lines following stimulation with soluble IL-15Rα

A-B Scatterplots showing the correlation between expression ratio (IL-15Rα-stimulated over control) of common significant deregulated proteins between primary and metastatic cell lines in each pair of melanoma cell lines, T1 and G1 (**A**), WM-115 and WM-266-4 (**B**). r and p values were determined by the Pearson correlation test. Linear regression and standard deviation are shown. **C** Venn diagram showing the intersection of significant deregulated proteins after IL-15Rα stimulation between the four primary and metastatic melanoma cell lines analysed.

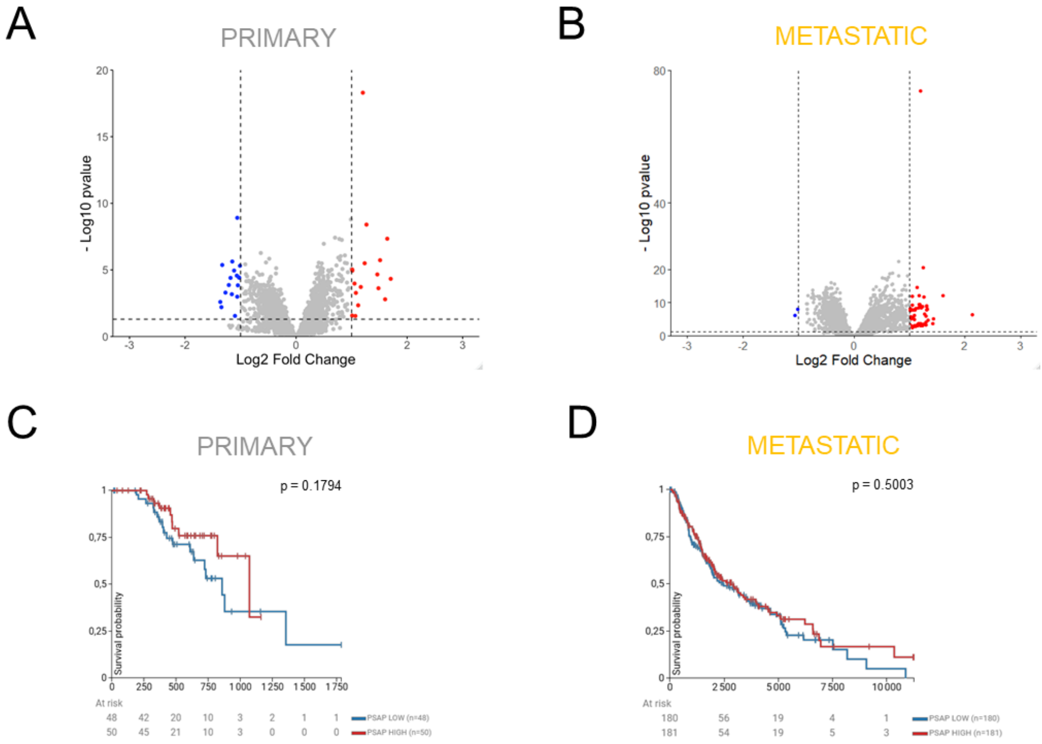

Figure Supplementary 3. Differential gene expression and survival analysis in primary and metastatic melanoma patients stratified by prosaposin expression levels

A-B Volcano plots of the differential expressed genes (DEG) in PSAP high versus PSAP low in primary (**A**) and metastatic (**B**) melanoma patients in the TCGA Melanoma SKCM data set. Samples were divided into categories of high and low PSAP expression by median as a cut-off value. Vertical lines indicate |log2 Fold Change|≥1 and horizontal line indicates p≤0,05 representing cut-off lines applied to filter significant DEG. Not significant DEGs are represented in grey, significant upregulated and downregulated DEGs are represented respectively in red or in blue. **C-D** Kaplan Meier curve showing the correlation between PSAP expression and overall survival of patients with primary (**C**) and metastatic (**D**) tumors from the TCGA SKCM data set. p values are shown.

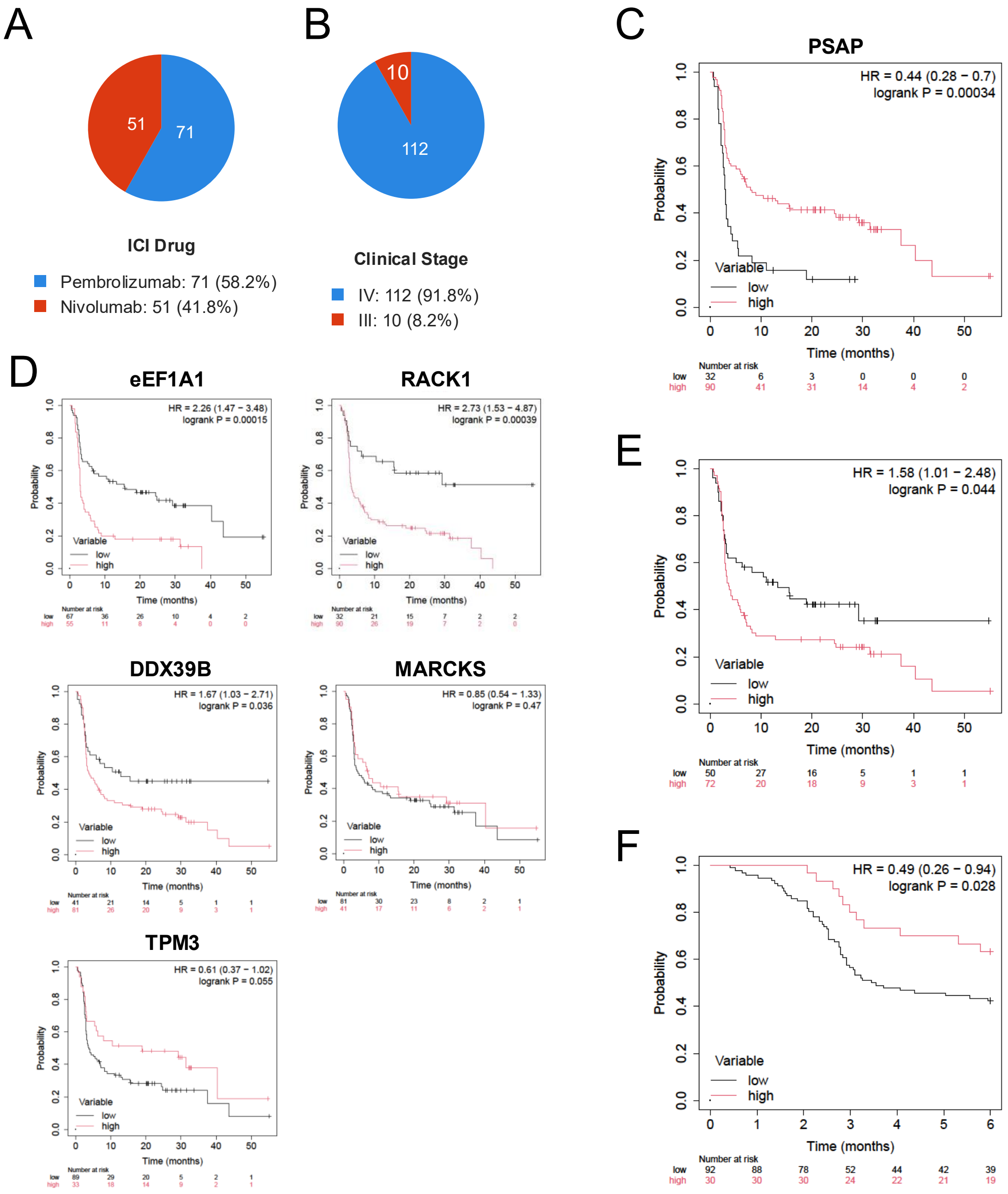

Figure Supplementary 4. Impact of the tmbIL-15 reverse signaling key factors on Progression-Free Survival in ICI-Treated Melanoma.

Metastatic Melanoma (DFCI, Nat Med. 2019) dataset was analyzed, including only ICI-treated patients. **A-B** Pie charts of the proportion of the ICI drugs (**A**) and of tumor stages distribution (**B**) in the cohort analyzed. **C-F** Kaplan Meier curve for progression-free survival stratified by the expression levels of PSAP (**C**), eEF1A1, RACK1, DDX39B, MARCKS, and TPM3 (**D**) and by the mean expression of PSAP, RACK1, EEF1A1, and DDX39B (**E**) and of PSAP, MARCKS, and TPM3 (**F**). Patients were stratified using the optimal percentile-based cut-off.
